# The Effect of Colistin Treatment on the Selection of Colistin-Resistant *Escherichia coli* in Weaner Pigs

**DOI:** 10.3390/antibiotics10040465

**Published:** 2021-04-20

**Authors:** Shahana Ahmed, Claus Hansen, Ane Laursen Dahlkilde, Ana Herrero-Fresno, Ken Steen Pedersen, Jens Peter Nielsen, John Elmerdahl Olsen

**Affiliations:** 1 Department of Veterinary and Animal Sciences, Faculty of Health and Medical Sciences, University of Copenhagen, 1870 Frederiksberg C, Denmark; shahana@sund.ku.dk (S.A.); xml718@alumni.ku.dk (A.L.D.); ahefr@sund.ku.dk (A.H.-F.); ken@sund.ku.dk (K.S.P.); jpni@sund.ku.dk (J.P.N.); 2 Danish Agriculture & Food Council, SEGES Pig Research Centre, 1609 Copenhagen V, Denmark; cha@seges.dk

**Keywords:** *E. coli*, colistin, antimicrobial resistance, pig diarrhea, commensal

## Abstract

The treatment of diarrhea in the postweaning period is a common reason for the use of antimicrobials in pig production, and *Escherichia coli* is the single most important causative agent for this condition. Colistin has recently been classified as a critically important antimicrobial for human health, as it is a last-resort drug against certain multi-drug-resistant Gram-negative bacteria. Therefore, the use of colistin has been significantly reduced in some countries, including Denmark. Despite this, the drug is still commonly used to treat diarrhea in pigs in many countries, and there is a need to understand the risks associated with this practice. We performed a prospective cohort study to investigate the effect of colistin treatment on the changes in the average minimum inhibitory concentration (MIC) in commensal *E. coli* in a pig herd where no colistin-resistant bacteria were detectable before treatment. One group of pigs was batch treated with colistin after the clinical observation of diarrhea, one group was batch treated with colistin approximately 10 days before the expected onset of diarrhea, and a control group was not treated with colistin but provided with nonantimicrobial antidiarrheal feed supplement. Treatment with colistin in the dose and time combinations used did not result in a significant increase in the average colistin MIC values in *E. coli.* Moreover, no *E. coli* strains showed a MIC above the breakpoint of >2 mg/L against colistin. Co-selection of resistance to other antimicrobials was not observed.

## 1. Introduction

Antimicrobial resistance (AMR) is considered a threat to human health. Worldwide, at least 700,000 people die annually from infection caused by AMR bacteria, and if appropriate actions are not taken, it has been estimated that by the year 2050, this number may reach up to 10 million, and the cost will be up to USD 100 trillion annually [1]. In veterinary medicine, AMR causes an increased cost of treatment and threatens the wellbeing of animals. Livestock can further be a source of AMR to humans via the food chain, environment, and by direct or indirect contact [2,3], and therefore, it is important to investigate AMR in farmed animals [4].

Diarrhea in nursery pigs is frequently seen in piglets throughout the world until three weeks after weaning [5] and accounts for a major part of the overall antimicrobial consumption in the pig industry [6]. Treatment is, however, considered necessary in most cases when postweaning diarrhea occurs to reduce mortality and maintain the desired growth. Colistin has been used extensively for the treatment of diarrhea caused by *E. coli* in weaner pigs, and for several decades, it has been a frequent choice of drug for this indication in pigs [7,8]. In humans, the emergence of multi-drug-resistant Gram-negative bacteria, for which colistin was the only effective drug, and the emerging evidence of plasmid-mediated colistin resistance [9] led to the strict regulation on the use of this drug in Europe and a general call by the World Health Organization (WHO) to reserve the drug for human use. Worldwide, however, colistin is still commonly used for the treatment of diarrhea in pigs, and it is important to know how this affects resistance development. Therefore, the aim of this work was to determine how different treatment regimens with colistin affects the resistance levels toward the drug in commensal *E. coli* from nursery pigs in an environment where resistance is low or absent before treatment, and to what extent the selection with colistin causes co-selection of resistance to other drugs.

## 2. Results

### 2.1. Effect of Treatment with Colistin at Clinical Indication or at a Fixed Timepoint before Expected Clinical Signs on the Selection of Colistin-Resistant Coliform Bacteria

**Figure 1:**
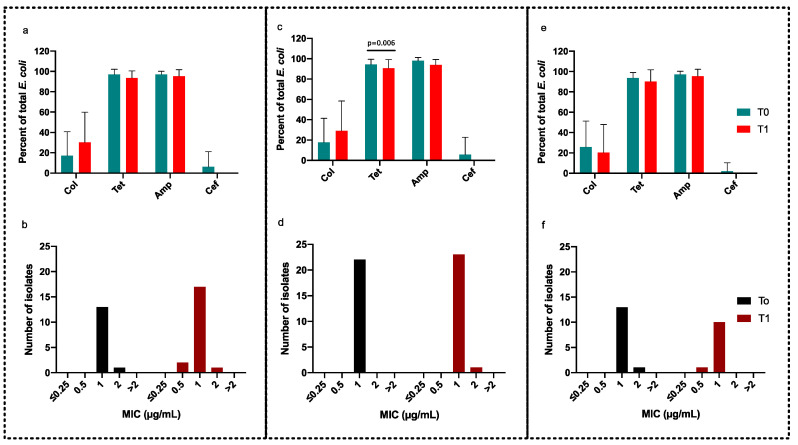
Percentage of resistant strains out of total *E. coli* and the MIC of *E. coli* against colistin in the different treatment groups. Treatment of pigs with colistin sulfate after the onset of diarrhea (**a**,**b**). Treatment of pigs with colistin sulfate before the onset of diarrhea (**c**,**d**). Treatment of pigs with antidiarrheal feed supplements (**e**,**f**). T0 and T1 refer to counts before and after treatment. Col, Tet, Amp, and Cef refer to CFU count on MacConkey agar plates supplemented with colistin, tetracycline, ampicillin, and cefotaxime, respectively. The error bars represent the standard deviation of the mean.

Two groups of pigs (G1 and G2) received oral treatment of colistin. In G1, the animals received the colistin treatment when the farmer evaluated that number of diarrheal droppings exceeded a pre-fixed level. In G2, the animals were treated 10 days before the expected onset of diarrhea. Presumptive colistin-resistant *E. coli* colonies were detected in both groups on the colistin-containing MCA plates before and after treatment (Table 1). As colistin resistance in *E. coli* is rare in Danish pigs, it was interesting to see whether the presumptive colistin-resistant *E. coli* isolated (i.e., colonies growing on MCA with colistin at the breakpoint concentration) (*n* = 105) from these two groups were truly resistant. The MICs of these *E. coli* isolates were determined by the BMD method, and detection of plasmid-mediated colistin resistance genes was performed by multiplex PCR. However, MIC values were below 2 µg/mL for all *E. coli* isolates (Figure 1d,e). The average MIC of colistin was 1.07 and 1.05 in T0 and T1, respectively in G1. In G2, the average MIC was 1.00 and 1.04 at T0 and T1, respectively. In concordance with this, no *mcr* genes were identified by PCR analysis. Therefore, no true colistin-resistant strains were found in this study. 

The average log_10_ CFU *E. coli* counts per gram feces declined from 7.9 to 6.6 and from 8.0 to 6.5 in the G1 and G2 groups, respectively, after treatment with colistin (Table 1). There was no selection of other antimicrobial resistances associated with treatment with colistin; rather, the tetracycline- and ampicillin-resistant *E. coli* decreased after treatment (Figure 1a,b and Table 1). For tetracycline, the decrease was significant; however, both before and after treatment, the percentage of *E. coli* that was tetracycline resistant was above 80%. Cefotaxime-resistant coliforms were detected in low concentrations in G1 and G2 (0.5 ± 0.3 and 0.4 ± 0.2 log_10_ CFU/g feces, respectively, before the initiation of treatment (T0)), while there was no cefotaxime-resistant *E. coli* at T1 (Table 1).

### 2.2. Effect of Antidiarrheal Feed on the Selection of Resistant Coliform Bacteria

In G3, the pigs received only the antidiarrheal feed. Additionally, in this group, the total average log_10_ CFU per gram feces of coliforms decreased from 7.9 to 6.4 after administering the antidiarrheal feed supplement (Table 1). In parallel, the percentage of tetracycline- and ampicillin-resistant coliforms decreased at T1 (Figure 1c). The average log_10_ CFU of cefotaxime-resistant coliforms was 0.2 in T0, and no resistant colonies were found after the trial period. Moreover, presumptive colistin-resistant colonies on colistin-containing MacConkey agar plates declined from 2.0 to 1.3 log_10_ CFU after administering the antidiarrheal feed supplement (Figure 1c). As for the other groups, G1 and G2, true colistin-resistant strains were not detected based on the MIC against colistin (Figure 1f). 

### 2.3. Effect of Treatments on Daily Growth and Health

Based on the daily growth of pigs, the control group, G3, administered the antidiarrheal feed supplement, showed the best performance compared to groups with the treatment of pigs with colistin (G1 and G2) (Supplementary Table S1). The percentages of pigs that died or become sick were comparatively higher in Farm 1 than Farm 2 (Supplementary Table S1).

## 3. Discussion

The main objective of this clinical field trial was to compare the effect of colistin treatment on the commensal colistin-resistant *E. coli* in pig farms. Resistance development was compared with a nonantibiotic group, where pigs were fed a diet specifically designed to reduce diarrheal episodes. For the evaluation of resistance development, the CFU data obtained from before treatment (T0) were compared with CFU data 21 days after treatment (T1). This approach did not reveal what happened in the *E. coli* population right after treatment, but it allowed us to compare whether pigs subjected to different treatment regimens would leave the nursery unit with different levels of antimicrobial-resistant (tetracycline, ampicillin, cefotaxime, and colistin) *E. coli*. The study revealed that the number of resistant bacteria in all treatment groups was lower 21 days after treatment than at T0, and based on the counting of total coliforms, this appeared to be caused by an overall reduction in the *E. coli* population over the 21 days of the study period. This finding is in accordance with results from previous studies, where the effect of the antimicrobial outlasted two weeks, and after that, the resistance reverted to similar levels detected at the starting point [10,11]. Among possible reasons explaining this, it has been suggested that improved host immunity with age affects the composition of the intestinal flora [12]. 

Colistin treatment led to an increase in the number of colonies capable of growing on MCA plates supplemented with the breakpoint concentration of colistin, while this was not observed in the nontreated group, G3. However, all colonies tested showed MIC values below the EUCAST breakpoint, and none of them contained any plasmid-mediated colistin resistance genes. These results are in agreement with the surveillance reports from DANMAP, where no colistin resistance has so far been detected in *E. coli* coliforms from Danish pigs [13]. The selection of resistance genes depends on the presence of that gene in the population, and this could be the reason we did not see any selection of colistin resistance in this study. Based on our results, we suggest using a higher concentration of colistin sulfate (>4 µg/mL) in agar media in future studies for the initial screening of colistin-resistant *E. coli*. The group G3 was not a control group in the strict sense, as it was fed a different feed, and thus the comparison between groups is not fully accurate. It would be highly interesting to follow pigs that were not treated at all. However, this was not possible from an animal welfare point of view. 

The current study estimated that the proportion of tetracycline-resistant *E. coli* in pigs decreased in numbers from T0 to T1. Even though these pigs did not receive any tetracycline treatment at T0, they might have acquired this *E. coli* resistant population from the sows or the farm environment [10]. Additionally, this high initial tetracycline resistance observed (Table 1) represents the overall tetracycline-resistant *E. coli* population in Danish pigs [13]. The number of tetracycline-resistant *E. coli* in G1 and G2 was not statistically different from the numbers in the group fed an antidiarrheal feed, G3, suggesting that 21 days after treatment, levels of tetracycline-resistant bacteria were unaffected by the colistin treatments, too. The study of resistance was conducted on one farm. Farm variation in response to tetracycline treatment has been shown to be high [11], and this should be considered when evaluating the results of the current study. 

No selection for ampicillin- or cefotaxime-resistant *E. coli* occurred in any of the two groups treated with colistin. The presence of cefotaxime-resistant *E. coli* was noticed at the initial screening (T0); however, at the second sampling (T1), no cefotaxime-resistant *E. coli* was found in any of the groups. This could be related to the general reduction in coliforms with age, which, again, could be due to changes in the diet as well as the modification of gut microbiota after weaning [14]. A previous study, conducted before the current voluntary ban on the use of third- and fourth-generation cephalosporins in Danish pigs in 2010, reported that the prevalence of cefotaxime-resistant *E. coli* was common in pre-weaned pigs and decreased with age after weaning [15]. Additionally, in both groups administered colistin, the ampicillin-resistant *E. coli* population significantly lowered from the first sampling (T0) to the second sampling (T1), and the frequency of ampicillin-resistant *E. coli* was comparable to the tetracycline-resistant *E. coli* population. This could be explained by the fact that there might be a population of *E. coli* in pigs in Denmark that are resistant to both tetracycline and ampicillin, as suggested in a previous study on coliforms from Danish pigs [16]. A similar tendency, involving the reduction of total coliforms as well as of each population of resistant coliforms to ampicillin, tetracycline, and cefotaxime from T0 to T1, was noticed in the group where the pigs received the antidiarrheal feed.

From a treatment point of view, colistin should be among the most effective drugs, as the incidence of colistin-resistant *E. coli* is generally very low in Danish pigs, as shown in this study and previous reports in Denmark [7]. It was therefore surprising that the growth rate was higher in the group with improved feed, compared to the colistin-treated groups. Coupled with the fact that the number of sick and dead pigs did not differ significantly either, this suggests that improved feeding can be used as an alternative to antibiotic treatment for postweaning diarrhea in pigs. Further studies with more farms included are needed to fully investigate this, as farm variation in management factors may be considerable. In the current study, for example, morbidity and mortality among pigs were at the expected level in Farm 1 (Supplementary Table S1), while the number in Farm 2 was significantly lower than what is normally seen in Danish pig herds [17], despite treatments being the same in both farms. 

## 4. Materials and Methods

### 4.1. Clinical Field Trial

The clinical treatment study was conducted as a randomized, nonblinded prospective cohort. The study included a total of 288 pens from 5 nursery units in two Danish pig farms where postweaning diarrhea was a common problem. The vaccination of sows to prevent neonatal cases of *E. coli* infection was performed on sows giving rise to the piglets used in the study. On one farm, ten rotations were included per treatment group, and on another farm, six rotations in each treatment group were included. Diarrhea scoring was carried out in both farms, while the study of resistance development was carried out in one farm, involving 57 pens. The study duration was from April 2016 to February 2017. At the start of the trial, it was made sure that there was no ongoing outbreak of Porcine Reproductive and Respiratory Syndrome (PRRS), porcine edema disease, *Mycoplasma hyopneumoniae,* and *Actinobacillus pleuropneumoniae* infections in the herds, and that the herds had not been diagnosed with diarrhea caused by *Brachyspira hyodysenteriae* within the past year. Moreover, during the period of study, the pig herds were immunized against *Lawsonia intracellularis* with the oral vaccine (Enterisol^®^ Ileitis, Boehringer Ingelheim, GA, USA) immediately after transfer to the nursery stable. At least four previous batches of pigs from each of the nursery units were examined to determine the pathogen profile of the herd before the start of the study (fecal material from sock samples) using a published qPCR-based protocol for the detection of pathogens [18].

The trials were conducted on pigs raised in an ‘all-in all-out’ of the nursery unit system. Herd 1 bought pigs at weaning (4 weeks old). Herd 2 was an integrated production. This farm, too, practiced weaning at 4 weeks. Each nursery unit contained a batch of weaned pigs inserted on the same day. There were three different treatment groups. The first group (G1) received an oral batch treatment with 4.8 mg of colistin sulfate per kg of body weight daily for 5 days (Colicol, ScanVet Animal Health A/S, Fredensborg, Denmark). The medication was provided with drinking water. Batch treatment with antibiotics was initiated in this group when the farmer evaluated that, on average, there were 1.5 diarrheal droppings per pen [19] in the nursery unit. The second group (G2) received oral batch treatment with colistin sulfate as above as metaphylactic treatment when *E. coli* (F4, F18) was diagnosed, i.e., colistin sulfate was given approximately 10 days before the expected onset of diarrhea as determined from observations from previous batches of pigs (data not shown). Screening for the presence of pathogens (fecal sampling) was performed prior to treatments in this group, as described above, with a specific focus on the screening of the herd for an increase in the excretion of *E. coli* (F4, F18). The pigs from G1 and G2 were fed with commercially purchased pelleted feed supplemented with therapeutic zinc oxide (2500 ppm) for the first 14 days after weaning. No antibiotic was added to the feed mixture. The third group (G3) was provided with an antidiarrheal feed for 10 days, consisting of a specially designed feed supplement. This group, too, was given therapeutic zinc oxide (2500 ppm) for 14 days, and no antibiotic was added to the feed mixture (Supplementary Table S2) [20]. The feed ingredients and amount were chosen based on previous findings [21,22,23,24,25] in an attempt to minimize diarrhea in weaning pigs by including a low level of protein, highly digestible proteins, and additives such as organic acids. The pens were excluded from the trial if the farmer or veterinarian assessed that the animals should be therapeutically treated with other drugs and another dosage or time of treatment than the study prescribed. Animals with injuries were taken out of the study, and clinical records were documented for the removed animals.

### 4.2. Growth and Clinical Observations

Data on the growth of individual pigs were recorded 3–7 days after the pigs entered the nursery unit and again on Day 42. In addition, individual and batch treatments for diarrhea, dead pigs, and other health data were recorded at departure from the piglet barn.

### 4.3. Sampling for Resistance Determination

From each pen, a composite fecal sample was collected on a sterile sock, as described in a previous study [18], by walking through the pen just after the pigs entered the pen and before the start of the treatment (T0, approximately 5 days after weaning), and sampling was repeated on Day 21 after treatment, before the pigs left the nursery stable (end of weaning period, T1, approximately 42 days after weaning). The sock samples were placed in a polyethylene bag labeled with the sample identity and sent to the laboratory in the nonfrozen state in cooling boxes containing ice, where they arrived the following day. A total of 114 sock samples were analyzed in this study.

### 4.4. Bacterial Quantification

The fecal materials from each of the sock samples were scraped off carefully. One gram of fecal sample was diluted in 9 mL of phosphate-buffered saline (PBS) to make a 1:10 dilution, which was then tenfold serially diluted up to 10^−6^. Two technical replicates were prepared for each sample. Twenty microliters of each dilution were plated onto different agar plates by the drop plate method [26]. Quantitative determination of the total number of *Coliform bacteria* was carried out on MacConkey agar (MCA) (Oxoid, Thermo Scientific, Roskilde, Denmark), and the quantification of presumptive drug-resistant *Coliform bacteria* was carried out on MacConkey agar supplemented with 2 µg/mL colistin sulfate (MCA + Col), 16 µg/mL tetracycline (MCA + Tet), 16 µg/mL ampicillin (MCA + Amp) and 2 µg/mL cefotaxime (MCA + Cef) antimicrobials as previously reported [27,28]. The concentration of antimicrobials on the agar plates was based on breakpoints of resistant *E. coli*, recommended by the European Committee on Antimicrobial Susceptibility Testing (EUCAST) [29]. The agar plates were incubated overnight at 37 °C, and dark red, nonmucoid colonies larger than 0.5 mm were counted as presumptive *E. coli* colonies. For statistical purposes, the counted colony numbers were converted into log_10_ CFU per gram of feces.

All presumptive colistin-resistant *E. coli* colonies (*n* = 105) were collected and stored at −80 °C from the colistin-added agar plates for further analysis. The species identity of randomly picked colonies (*n* = 600) from MCA containing the different antibiotics was confirmed to be *E. coli* by matrix-assisted laser desorption ionization-time of flight mass spectrometry (MALDI-TOF MS; BioMérieux, France).

### 4.5. Minimal Inhibitory Concentration (MIC) Determination 

All the presumptive colistin-resistant *E. coli* (*n* = 105) colonies isolated from colistin-containing MacConkey agar plates were subjected to the broth microdilution (BMD) method to determine the MIC of colistin sulfate [30]. To standardize the method, a cation-adjusted Mueller–Hinton Broth II (Sigma-Aldrich, St Louis, MO, USA) was used. *E. coli* NCTC 13846 (colistin resistant) and *E. coli* ATCC 25922 (colistin susceptible) were used in all MIC experiments as the quality control strains.

### 4.6. Multiplex PCR for Plasmid-Mediated Genes for Colistin Resistance

A previously described method based on multiplex PCR to determine the presence of five plasmid-mediated *mcr* genes (*mcr-1*, *mcr-2*, *mcr-3*, *mcr-4*, and *mcr-5*) was used to screen all the presumptive colistin-resistant *E. coli* (*n* = 105) collected from MacConkey agar plates supplemented with colistin sulfate [31]. The positive reference controls for *mcr-1*, *mcr-2*, *mcr-3*, *mcr-4*, and *mcr-5* genes were *E. coli* NCTC 13846, *E. coli* KP37 [32], *E. coli* 2013-SQ352 [31], *E. coli* DH5α [33], and *Salmonella* 13-SA01718 [34], respectively. *E. coli* ATCC 25922 (colistin susceptible) was also included during PCR as a negative control. As an external reference control, GeneRuler 100 bp Plus DNA Ladder (Thermo Fisher Scientific, Roskilde, Denmark) was used in the first and last well of each gel.

### 4.7. Statistical Analysis

Changes in the log_10_ CFU of coliforms per gram of feces from before to after treatment were analyzed by a two-tailed unpaired parametric *t*-test in GraphPad Prism 8.1.1 (GraphPad Prism Software Inc., San Diego, CA, USA). The effect of the three different treatments on the growth of pigs was analyzed as the average daily growth with a linear mixed model with individual-level growth from the starting weight to the end-weight as a dependent variable. The explanatory variable in the model was the treatment group (G1–G3), while the pen, nursery unit, and rotation batch were included as a random effect. The weight of each individual pig when entering the nursery unit was included as the covariate in the model. The growth data were recently published in a report by SEGES (in Danish) [12], communicating the results to Danish pig farmers.

## 5. Conclusions

The current study has shown that treatment with colistin in the dose and time combinations used did not result in a significant increase in the average colistin MIC values in *E. coli* or co-resistance to other commonly used drugs to treat pigs. Surprisingly, an antidiarrheal feed to pigs around weaning was shown to result in higher weight gain than colistin treatment; however, further studies are needed to explain this observation. 

## Figures and Tables

**Table 1:** Analysis of the *E. coli* population in the different treatment groups.

Treatment Group	*E. coli* Population	Log_10_ CFU/gm Feces ± SEM (T0)	Log_10_ CFU/gm Feces ± SEM (T1)
G1 (Colistin after the onset of diarrhea)	Total	7.9 ± 0.1	6.6 ± 0.2
	Tetracycline-resistant	7.7 ± 0.2	6.2 ± 0.2
	Ampicillin-resistant	7.7 ± 0.2	6.3 ± 0.2
	Cefotaxime-resistant	0.5 ± 0.3	Not detected
	Presumptive colistin-resistant *	1.4 ± 0.4	1.9 ± 0.42
G2 (Colistin before the onset of diarrhea)	Total	8.0 ± 0.2	6.5 ± 0.2
	Tetracycline-resistant	7.5 ± 0.2	5.9 ± 0.2
	Ampicillin-resistant	7.8 ± 0.1	6.1 ± 0.2
	Cefotaxime-resistant	0.4 ± 0.3	Not detected
	Presumptive colistin-resistant *	1.4 ± 0.4	1.8 ± 0.4
G3 (nonantimicrobial antidiarrheal feed supplement)	Total	7.9 ± 0.2	6.4 ± 0.2
	Tetracycline-resistant	7.4 ± 0.2	5.7 ± 0.2
	Ampicillin-resistant	7.6 ± 0.2	6.1 ± 0.2
	Cefotaxime-resistant	0.2 ± 0.2	Not detected
	Presumptive colistin-resistant *	2.0 ± 0.5	1.3 ± 0.4

CFU, colony-forming unit; SEM, standard error of the mean. T0 is counts at weaning and before the start of treatment; T1 is counts 21 days after weaning and after the end of treatment. * Based on the growth of *E. coli* on MacConkey agar supplemented with 2 µg/mL colistin sulfate.

## Data Availability

All data supporting the reported results are presented as Tables, Figures, or as a supplementary document in this manuscript.

## Supplementary Materials

The following are available online at https://www.mdpi.com/article/10.3390/antibiotics10040465/s1, Supplementary Table S1: Overview of animals included and results for pig growth and the number of herd treatments at the pen level and the number of treatments started per group in both farms. Supplementary Table S2: Feed composition used for the experimental animals.
