# Supplementary material for: The Effect of Colistin Treatment on the Selection of Colistin-Resistant Escherichia coli in Weaner Pigs
Source: Antibiotics (Basel). 2021 Apr 20;10(4):465. doi: 10.3390/antibiotics10040465 (PMC8073783; doi:10.3390/antibiotics10040465)
Supplement: Supplementary file 1 [file antibiotics-10-00465-s001.zip › Supplementary_Table2.docx]

**Supplementary Table 2.** Feed composition used for the experimental animals.

1. Composition of feed mixture for group 1 and 2.

| **Raw materials (%)** | **Mixture 1**  **6-10 kg**  **All groups**  **April-August** | **Mixture 1**  **6-10 kg**  **All groups**  **August-October** | **Mixture 2**  **10-13 kg**  **Group 1 and 2** | **Mixture 3**  **13-20kg**  **All groups** | **Mixture 4**  **20-30 kg**  **All groups** |
| --- | --- | --- | --- | --- | --- |
| Barley | 31.5 |  | 27 | 18 | 20 |
| Wheat | 31.8 |  | 42.8 | 52.1 | 47.2 |
| Fishmeal | 4.2 |  | 6 | 5 | - |
| Soy diets |  |  | 17 | 18 | 26.2 |
| Fat | 2.5 |  | 2.5 | 2.2 | 2 |
| B2B-m-a-01 Conc 30% | 30 |  | - | - | - |
| Mineral feed mixture VitFoss | - |  | 4.7 | 4.7 | 4.6 |
|  |  |  |  |  |  |
| Ingredient contribution from concentrate (%) | | | |  |  |
| Corn | 0.33 | 1.74 |  |  |  |
| Oats | 2.1 | 2.1 |  |  |  |
| Milk powder | 7.5 | 7.5 |  |  |  |
| Potato | 1.42 | 2.61 |  |  |  |
| Protein concentrate | 1.42 | 0.00 |  |  |  |
| Encellet Protein | 12.4 | 11.07 |  |  |  |
| Soy protein concentrate | 0.46 | 0.49 |  |  |  |
| L-Lysine hydrochloride | 0.1 | 0.11 |  |  |  |
| DL-Methionine | 0.01 | 0.19 |  |  |  |
| L-Threonine | 0.13 | 0.13 |  |  |  |
| Tryptophan | 0.04 | 0.07 |  |  |  |
| Valin | 1.36 | 1.36 |  |  |  |
| Chalk | 1.23 | 1.16 |  |  |  |
| Monocalcium phosphate | 0.41 | 0.38 |  |  |  |
| Feed salt | 0.54 | 0.54 |  |  |  |
| Premix | 0.05 | 0.05 |  |  |  |
| Enzyme – FYT | 0.00 | 0.00 |  |  |  |
| Enzyme - Xylanase | |  |  |  |  |
| Taste and aroma substance | 0.01 | 0.01 |  |  |  |
| Vitamin | 0.49 | 0.49 |  |  |  |
| Benzoic acid | 0.5 | 0.5 |  |  |  |

B. Composition of feed mixture for group 3.

| **Raw materials** | **(%)** |
| --- | --- |
| Barley | 0 |
| Wheat | 0 |
| Leci Grain Heat Treated Cross | 55.74 |
| Oat flakes / groats | 5.00 |
| Kagemix | 3.17 |
| Fishmeal | 2.00 |
| HP 800 | 6.82 |
| Potato protein conc. | 1.50 |
| Plasma Appetein | 4.50 |
| Yogurt milk | 3.00 |
| Whey powder | 14.00 |
| Lysine | 0.449 |
| Methionine | 0.145 |
| Threonine | 0.015 |
| Tryptophan | 0.061 |
| Mono Calcium Phosphate | 0.85 |
| Feed salt | 0.10 |
| Vitamin (VA) | 0.308 |
| Vitamin E (VA) | 0.32 |
| Na Buthyrat | 0.10 |
| Sucram TikTak | 0.015 |
| Aroma (butter/vanilla) | 0.02 |
| Ronozyme NP | 0.015 |
| Bactocell MD | 0.01 |
| Diamol DI 2000 | 0.30 |
| Xylanase | 0.05 |
| OV11 Conc | 0.002 |
| Benzoic acid (Vevovitall) | 0.50 |
| Calcium format | 1.006 |

(The table is adapted from the report (in Danish) conducted by Hansen et al., 2018.)
