# Supplementary material for: The Effect of Colistin Treatment on the Selection of Colistin-Resistant Escherichia coli in Weaner Pigs
Source: Antibiotics (Basel). 2021 Apr 20;10(4):465. doi: 10.3390/antibiotics10040465 (PMC8073783; doi:10.3390/antibiotics10040465)
Supplement: Supplementary file 1 [file antibiotics-10-00465-s001.zip › Supplementary_Table1.doc]

**Supplementary Table 1. Overview of animals included and results for pig growth and the number of herd treatments at the pen level and the number of treatments started per group in both farms.**

|  | **Farm 1** |  |  | **Farm 2** |  |  |
| --- | --- | --- | --- | --- | --- | --- |
| Treatment group | **G1** | **G2** | **G3** | **G1** | **G2** | **G3** |
| Number of rotations | 10 | 10 | 10 | 6 | 6 | 6 |
| Number of pens | 36 | 36 | 36 | 36 | 36 | 36 |
| Total number of pigs per pen per rotation at insertion | 40 | 40 | 40 | 30 | 30 | 30 |
| Number of pigs weighed | 540 | 539 | 540 | 540 | 539 | 541 |
| Average weight at the beginning (approximately 5 days after weaning) (kg) | 8.90 | 9.15 | 8.92 | 7.55 | 7.62 | 7.57 |
| Average weight at the end (approximately 42 days after weaning) (kg) | 30.43 | 31.67 | 33.21 | 23.61 | 24.22 | 25.02 |
| Percentage of pigs with normal weight at the beginning (%) | 95.2 | 97.4 | 98.2 | 81.7^a^ | 85.6^a^ | 90.6^b^ |
| Percent died and taken out for treatment | 6.11 | 4.64 | 3.52 | 2.04 | 1.11 | 0.92 |
| Average Daily Growth (g) (LSMEANS) | 533^b^ | 539^b^ | 590^a^ | 428^b^ | 438^b^ | 461^a^ |
| Average number of treatments started (individual treatments + herd treatments) | 1.33 | 1.71 | 0.88 | 1.34 | 1.74 | 1.79 |

Different superscripts indicate a statistically significant difference (*p*<0.05). G1, treatment of pigs with colistin sulfate after the onset of diarrhea. G2, treatment of pigs with colistin sulfate before the onset of diarrhea. G3, treatment of pigs with anti-diarrheal feed supplement before the onset of diarrhea. (The table is adapted from the report (in Danish) conducted by Hansen et al., 2018.)
